# Puerperal Eclampsia Treated by Veratrum Viride

**Published:** 1884-11

**Authors:** 


### Puerperal Eclampsia Treated by Veratrum Viride.

Dr. H. W. Brown, of Hillsboro, Ohio, reports six cases of puerperal eclampsia, in the *Obstetric Gazette*, treated entirely by Norwood's tinct. of veratrum viride. He gives it in doses of from forty to ninety drops every few minutes, till vomiting, relaxation and a fall of the pulse, to or below normal, occurs.

Theoretically, it is indicated in those cases in which venesection was formerly advised by the older obstetricians. Dr. Brown closes his article by saying that these cases treated thus show that the fears entertained by the profession as to its deadly poisonous effects, when administered in large doses, is wholly groundless.

---

Dr. John B. Roberts, of Philadelphia, in the *Polyclinic* of Sept. 15, 1884, expresses himself in favor of more frequent trephining in cranial fractures. While this line of treatment is more heroic than is generally taught, still it is thought it will commend itself to those who will carefully consider the subject. Each case must, of course, be studied by itself, and the patient's chances of death, of life with subsequent epilepsy or insanity, or of return to perfect health, carefully weighed. As a general guide, however, for the student and practitioner, Dr. Roberts presents the following table, which, we think, will be found, in the main, correct:

